# Ras associated with diabetes may play a role in fracture nonunion development in rats

**DOI:** 10.1186/s12891-019-2970-9

**Published:** 2019-12-12

**Authors:** Takahiro Oda, Takahiro Niikura, Tomoaki Fukui, Michio Arakura, Keisuke Oe, Yutaka Mifune, Shinya Hayashi, Tomoyuki Matsumoto, Takehiko Matsushita, Ryosuke Kuroda

**Affiliations:** Department of Orthopedic Surgery, Kobe University Graduate School of Medicine, 7-5-1, Kusunoki-cho, Chuo-ku, Kobe, 650-0017 Japan

**Keywords:** Rad, Rem1, Nonunion, Fracture

## Abstract

**Background:**

Rad is the prototypic member of a subfamily of Ras-related small G-proteins and is highly expressed in the skeletal muscle of patients with type II diabetes. Our previous microarray analysis suggested that Rad may mediate fracture nonunion development. Thus, the present study used rat experimental models to investigate and compare the gene and protein expression patterns of both Rad and Rem1, another RGK subfamily member, in nonunions and standard healing fractures.

**Methods:**

Standard healing fractures and nonunions (produced via periosteal cauterization at the fracture site) were created in the femurs of 3-month-old male Sprague-Dawley rats. At post-fracture days 7, 14, 21, and 28, the fracture callus and fibrous tissue from the standard healing fractures and nonunions, respectively, were harvested and screened (via real-time PCR) for *Rad* and *Rem1* expression. The immunolocalization of both encoded proteins was analyzed at post-fracture days 14 and 21. At the same time points, hematoxylin and eosin staining was performed to identify the detailed tissue structures.

**Results:**

Results of real-time PCR analysis showed that *Rad* expression increased significantly in the nonunions, compared to that in the standard healing fractures, at post-fracture days 14, 21, and 28. Conversely, immunohistochemical analysis revealed the immunolocalization of Rad to be similar to that of Rem1 in both fracture types at post-fracture days 14 and 21.

**Conclusions:**

Rad may mediate nonunion development, and thus, may be a promising therapeutic target to treat these injuries.

## Background

Nonunions represent a challenging complication of bone fractures. In fact, it is estimated that 5–10% of all fractures fail to heal normally, resulting in delayed union or nonunion (although this rate varies in patients with diverse medical histories) [1, 2]. Despite recent advances in orthopedic research, many aspects of the pathophysiology and molecular basis of nonunions remain unclear [3, 4].

We have previously investigated and compared gene expression patterns in nonunions and standard healing fractures, using rat experimental models. In that study, the fracture callus and fibrous tissue of the standard healing fractures and nonunions, respectively, were harvested and subjected to a microarray analysis. Results of this analysis indicated that the expression of *Ras associated with diabetes* (*Rad, RRAD*) was higher in nonunion tissues than in fracture calluses in a standard fracture-healing model [3]. Nevertheless, the specific function of Rad in fracture healing and/or nonunion development is still unknown.

Rad is a prototypic member of a subfamily of Ras-related small G-proteins [5, 6] that have been implicated in a wide range of cellular processes, including cell growth and differentiation [7–9]. Although Rad has been reported to be highly expressed in the muscle of patients with type II diabetes [10] and to be associated with both vascular lesion formation and cardiac disease [11–13], it has not been focused upon in bone biology research. In fact, to the best of our knowledge, our previous report was the first to investigate the role of *Rad* in fracture healing or nonunion development.

To test the hypothesis that Rad may mediate nonunion development, the present study used rat experimental models to investigate *Rad* gene and protein expression patterns in both nonunion fibrous tissue and in fracture calluses harvested from standard healing fractures. The patterns were further compared to those of another G-protein family member, *rad and gem related GTP binding protein 1* (*Rem1*, *REM1*) [14]. We also investigated the gene expression patterns of *tumor necrosis factor-α (TNF-α)* and *platelet-derived growth factor (PDGF)*, which are considered involved in the regulation of Rad gene expression [11].

## Methods

### Animals

Fifty-two 3-month-old male Sprague-Dawley rats (SLC Japan, Shizuoka, Japan) were used in this study, and all experiments were performed under the approval and guidance of the Animal Care and Use Committee of Kobe University Graduate School of Medicine (Approval Number: P170702). Animals randomly received surgical treatment to either establish a nonunion (*n* = 26), or to produce a standard stabilized closed femoral shaft fracture that is known to successfully heal, as previously described (n = 26) [15–17]. Briefly, standard healing fractures were produced via the retrograde insertion of a 1.25 mm-diameter K-wire into the right femoral intramedullary canal and induction of a closed transverse femoral shaft fracture using a three-point bending apparatus using a drop weight [15]. After creating the fracture, we repaired the patellar aponeurosis and the skin using nylon sutures. To produce a nonunion, after producing the fracture model as mentioned above, the fracture site was minimally exposed using a lateral approach, and the circumference of the periosteum was cauterized to a depth of 2 mm on both sides of the fracture, as previously reported [16]. After treatment, the damaged tissues were repaired in the same manner as performed for standard healing fractures, and the fascia and skin of the lateral thigh wound were closed using nylon sutures. Preoperatively, we injected medetomidine (0.15 mg/kg), midazolam (2 mg/kg), and butorphanol (2.5 mg/kg) intraperitoneally for anesthesia and sedation. Postoperatively, we also injected benzylpenicillin potassium (100 thousand units/kg) intraperitoneally as an antibiotic agent. Unprotected weight bearing was allowed postoperatively.

From five animals from each group at post-fracture days 7, 14, 21, and 28, newly generated specific tissues (i.e., fracture calluses from the standard healing fractures, and fibrous tissue from the area surrounding the nonunion fracture sites) were carefully and selectively harvested by circumferential excision from the underlying intact cortical bone, to provide material for gene expression analysis. Femurs were harvested from three animals from each group at post-fracture days 14 and 21 to provide materials for the immunohistochemical analysis. At each time points of assessment, all animals were euthanized by an overdose of pentobarbital sodium intraperitoneally.

### RNA extraction

Tissues were homogenized using TRIzol (Invitrogen, Carlsbad, CA) with a T18 ULTRA-TURRAX homogenizer (IKA Werke, Staufen, Germany) immediately after harvesting. Total cellular RNA was extracted from the harvested tissue using the acid guanidium thiocyanate-phenol-chloroform method and purified, first using a RNeasy Mini Kit (Qiagen, Valencia, CA) and then via column-digestion using an RNase-free DNase kit (Qiagen) (to prevent contamination with genomic DNA).

### Real-time polymerase chain reaction

**Fig. 1:**
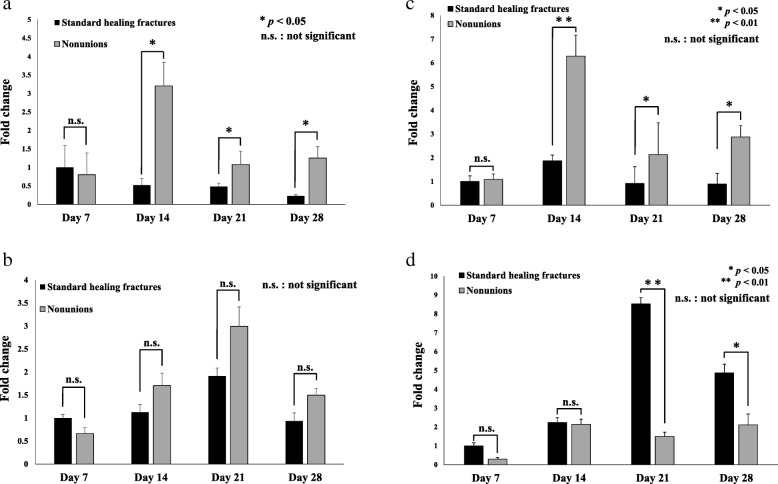
**a** *Rad*, **b** *Rem1*, **c** *TNF-α*, and **d** *PDGF* expression in standard healing fractures (black bars) and nonunions (gray bars) at post-fracture days 7, 14, 21, and 28, as analyzed by real-time PCR. All graphs show the fold change and range for each experimental group, normalized to the gene expression levels observed in standard healing fractures at post-fracture day 7. *p < 0.05 and **p < 0.01 indicate statistically significant difference between standard healing fractures and nonunions at the indicated time points

RNA samples were reverse-transcribed to synthesize single-stranded cDNA samples using a High Capacity cDNA Reverse Transcriptional kit (Applied Biosystems, Foster City, CA) according to the manufacturer’s instructions. We purchased TaqMan primers and probe pairs for *Rad*, *Rem1*, and glyceraldehyde-3-phosphate dehydrogenase (*GAPDH*, internal control) and SYBR Green primers for *TNF-α*, *PDGF*, and *GAPDH* (primer sequences are shown in Table 1) from Applied Biosystems. Real-time PCR was performed using the ABI 7700 Sequence Detector (Applied Biosystems), according to the manufacturer’s instructions. *Rad*, *Rem1*, *TNF-α,* and *PDGF* expression levels were normalized to those of *GAPDH* and expressed relative to gene expression levels observed in the standard healing fractures on post-fracture day 7 (∆∆CT methods; Applied Biosystems) [18]. All graphs (Fig. 1a, b) demonstrate the gene expression as a fold change relative to the average gene expression values (normalized to 1) observed in the standard healing fractures at post-fracture day 7.

**Table 1:** Specific primers used for real-time PCR amplifications Gene name Primer sequence (5′-3′)

Gene name	Gene name Primer sequence (5′-3′)	
	Forward	Reverse
GAPDH	AAATGGTGAAGGTCGGTGTG	TGAAGGGGTCGTTGATGG
TNF-α	GCTCACAATGTCTGTGCTTAGAG	GCAGTAGCCACAGCTCCAG
PDGF	GTCCAGGTGAGGTTAGAGG	CACGGAGGAGAACAAAGAC

GAPDH; glyceraldehyde-3-phosphate dehydrogenase, TNF-α; tumor necrosis factor-α, PDGF; plateletderived growth factor

### Histology processing

Harvested femurs were fixed in 4% paraformaldehyde at room temperature for 24 h. Next, femurs were decalcified at room temperature with a decalcifying solution (composition: 10% formic acid and 10% formalin, the ratio was 1: 1) and embedded in paraffin wax. Finally, the femurs were processed to produce 6-μm-thick sagittal sections using a microtome.

### Hematoxylin and eosin (H&E) staining

The sections were deparaffinized in xylene, dehydrated with graded alcohols, and stained with hematoxylin and eosin to determine the detailed histological structure.

### Immunohistochemistry

The deparaffinized sections were digested with target retrieval solution (Dako North America, Inc., Carpinteria, CA) for 20 min, and treated with 3% hydrogen peroxide (WAKO Pure Chemical Industries, Ltd., Osaka, Japan) to block endogenous peroxidase activity. The sections were incubated overnight at 4 °C with a Rad goat polyclonal (1:100 dilution), or Rem1 mouse monoclonal antibody (1:200) (Santa Cruz Biotechnology, TX), and subsequently treated with peroxidase-labeled anti-goat (Histofine Simplestain max PO (G), Nichirei Bioscience, Tokyo, Japan) and anti-rabbit immunoglobulin (Histofine Simplestain max PO (M), Nichirei Bioscience) at 25 °C for 60 min. The signal was developed using peroxidase substrate 3-amino-9-ethylcarbazole (Histofine Simplestain AEC Solution, Nichirei Bioscience), which yielded a brown reaction product. The sections were counterstained with methyl green and examined under the microscope.

### Statistical analysis

Fold changes in mRNA expression, calculated for the standard healing fractures and nonunions, were compared at each time point by applying Mann-Whitney U-test. A *p* value < 0.05 was defined as statistically significant. Columns and error bars indicate the mean and standard error, respectively (*n* = 5 per group).

## Results

### Rad and Rem1 gene expression in nonunions and standard healing fractures

No significant difference in *Rad* gene expression was observed between the standard healing fractures and nonunions at post-fracture day 7; however, *Rad* expression was significantly (6.2, 2.2, and 5.6-fold) higher in the nonunions than in the standard healing fractures at the later time points, i.e., post-fracture days 14, 21, and 28, respectively (Fig. 1a). Notably, *Rad* expression peaked in the standard healing fractures at post-fracture day 7, before declining, but continued to increase in the nonunions until day 14.

In contrast, no significant difference in *Rem1* expression was observed between the standard healing fractures and nonunions at any of the analyzed time points (Fig. 1b); *Rem1* expression did increase in the nonunions until post-fracture day 21.

### TNF-α and PDGF gene expression in nonunions and standard healing fractures

*TNF-α* gene expression was significantly (3.4-, 2.3-, and 3.2-fold) higher in the nonunions than in the standard healing fractures at post-fracture days 14, 21, and 28, respectively (Fig. 1c). This result was very similar to the gene expression pattern of *Rad*. In contrast, *PDGF* gene expression was significantly (5.7- and 2.3-fold) higher in the standard healing fractures than in the nonunions at post-fracture days 21 and 28, respectively (Fig. 1d).

### Rad and Rem1 protein expression in standard healing fractures

**Fig. 2:**
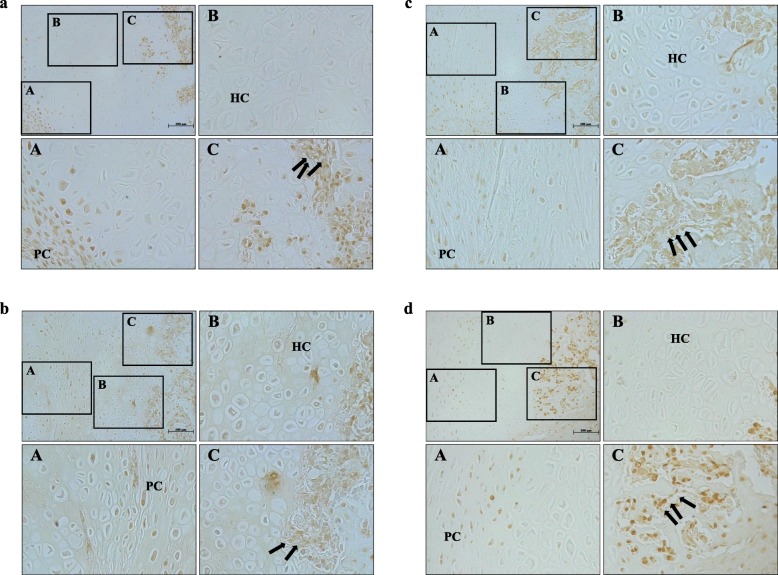
Immunohistochemical analysis showing Rad and Rem1 expression (brown staining) in cartilage calluses in standard healing fractures, as visualized using antibodies to **a** Rad at post-fracture days 14 and **c** 21, and **b** Rem1 at post-fracture days 14 and **d** 21. Bars, 100 μm; HC, hypertrophic chondrocytes; PC, proliferating chondrocytes. Black arrows, osteoblasts lining the trabecular bone surface

**Fig. 3:**
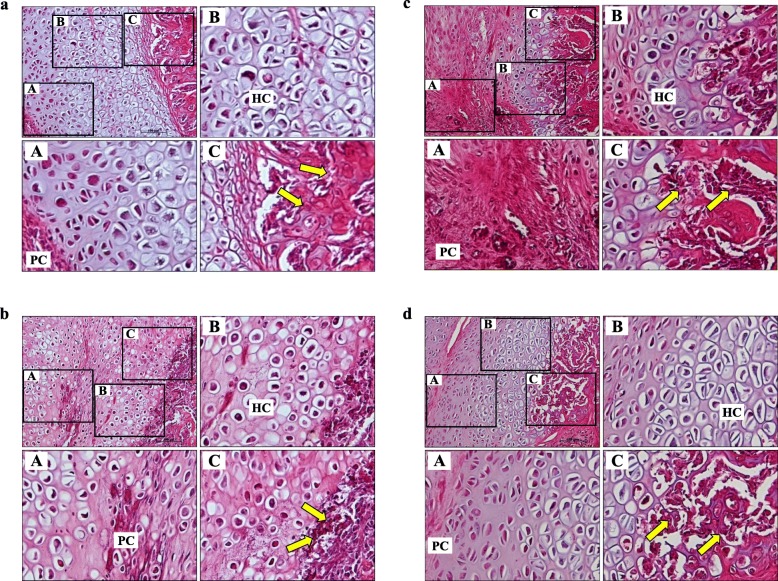
Histology of H&E staining of the cartilage callus in standard healing fractures at nearby sections of Fig. 2. Images of (a), (b), (c), and (d) correspond to (a), (b), (c), and (d) in Fig. 2, respectively. Bars, 100 μm. Yellow arrows, osteoblasts lining the trabecular bone surface.

On post-fracture day 14, the spaces between the newly formed callus in the standard healing fractures were filled with fibroblast-like spindle cells, rich proliferating chondrocytes, mature hypertrophic chondrocytes, and newly formed woven bone. Both intramembranous ossification and endochondral ossification were observed. Rad and Rem1 immunoreactivity was detected at robust levels in the fibroblast-like spindle cells, proliferating chondrocytes, and osteoblasts lining the trabecular bone, but only at very low levels in the hypertrophic chondrocytes. These patterns of Rad and Rem1 expression were maintained in cartilage calluses on post-fracture day 21. Notably, the regions of proliferating and hypertrophic chondrocytes were observed to decrease over time (Fig. 2, Fig. 3, Table 2).

**Table 2:** Summary of immunolocalization of Rad and Rem1

	Day 14		Day 21	
	Rad	Rem1	Rad	Rem1
Standard healing fractures				
Proliferating chondrocytes	+	+	+	+
Hypertrophic chondrocytes	–	–	–	–
Osteoblasts	+	+	+	+
Nonunions				
Fibroblast-like spindle cells	+	+	+	+

**-**, no staining; **+**, positive staining

### Rad and Rem1 protein expression in nonunions

**Fig. 4:**
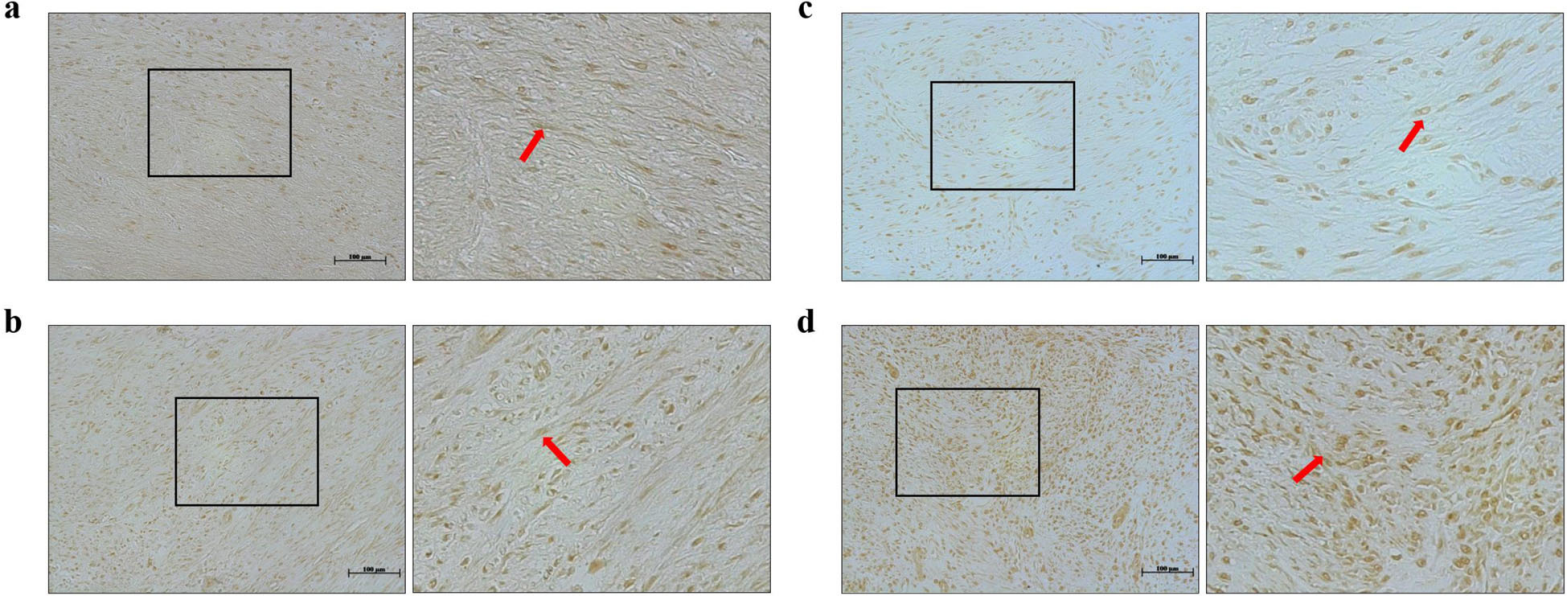
Immunohistochemical analysis showing Rad and Rem1 expression (brown staining) in nonunion fibrous tissue, as visualized using antibodies to **a** Rad at post-fracture days 14 and **c** 21, and **b** Rem1 at post-fracture days 14 and **d** 21. Bars, 100 μm. The fracture gap was covered by fibrous tissue filled with fibroblast-like spindle cells (red arrows), which exhibited positive Rad and Rem1 staining.

**Fig. 5:**
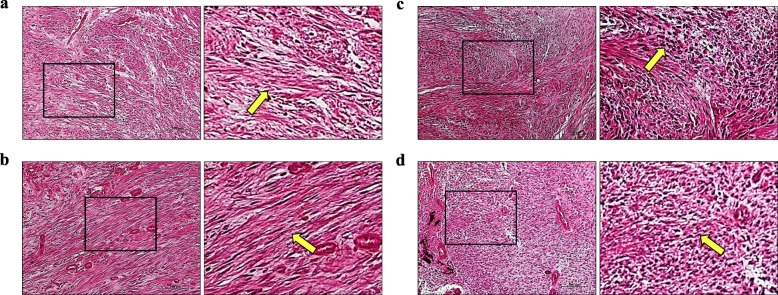
H&E staining of nonunion fibrous tissue at the nearby sections of Fig. 4. Images of (a), (b), (c), and (d) correspond to nearby sections of (a), (b), (c), and (d) in Fig. 4, respectively. Bars, 100 μm. Yellow arrows, fibroblast-like spindle cells

In the nonunions, the fracture gap was covered by fibrous tissue filled with fibroblast-like spindle cells. On post-fracture days 14 and 21, fibroblasts in the fibrous tissue expressed both Rad and Rem1. There was no difference in the patterns of staining and immunolocalization of Rad and Rem1 in nonunions and was no change over time in the positively stained area of both proteins (Fig. 4, Fig. 5, Table 2).

## Discussion

Rad and other Ras superfamily members have been shown to be activated by diverse extracellular stimulating factors and have been reported to be involved in a wide spectrum of cellular functions, including cell proliferation, differentiation, morphology, and apoptosis [19]. *Rad* was initially identified via subtractive cloning as an mRNA that is overexpressed in the skeletal muscle of a subset of patients with type II diabetes; however, it is normally highly expressed in the heart and lungs [10]. Previous studies have shown that Rad is regulated by TNF-α and PDGF [11] and that it interacts with skeletal muscle β-tropomyosin and the cytoskeleton of muscle cells [5] to inhibit insulin-stimulated glucose uptake in a variety of cultured cell lines [20]. Another study reported that Rad is temporarily regulated within myogenic progenitor cells during skeletal muscle regeneration [21]. Together, these reports suggest that Rad may mediate skeletal muscle function and regeneration, and/or cytoskeletal organization. Till date, however, its expression and role(s) in fracture healing and nonunion development have not been elucidated.

The RGK subfamily of small GTP-binding proteins comprises of four members, namely, Rad, Rem1, Rem2, and Gem (mouse homolog, also referred to as Kir) [22]. Of these, the present study selected Rem1 as a comparative protein for reference while investigating the relevance of Rad in nonunion development. Real-time PCR showed that, at the earliest analyzed time point (i.e., post-fracture day 7), there was no significant difference in *Rad* mRNA expression between the standard healing fractures and nonunions; however, *Rad* expression was significantly higher in the nonunions than in the standard healing fractures at later time points (i.e., post-fracture days 14, 21, and 28). In contrast, there was no significant difference in *Rem1* gene expression between the two treatment groups at any of the analyzed time points. This suggests that *Rad*, but not *Rem1*, may be important in nonunion development. Notably, the time points at which *Rad* expression was increased in the nonunions coincided with angiogenesis progression and endochondral ossification initiation in the current rat fracture model, thereby suggesting that *Rad* may impact or modulate these processes to mediate nonunion development. Further research is needed to examine the underlying mechanism(s) and significance of the observed increase in *Rad* expression in nonunions (compared to standard healing fractures) at the later time points.

Till date, Rad has been the focus of significant research in a range of contexts. For example, Fu et al. previously published a cardiology study showing that Rad is a critical inhibitor of vascular lesion formation, which acts by suppressing vascular smooth muscle cell migration [11]. Conversely, previous oncology studies have suggested that Rad inactivation may promote both hepatocellular carcinoma metastasis and nasopharyngeal carcinoma development [23, 24]. Another recent report suggested that Rad may play an important role in the regulation of bone homeostasis [25]; however, to our knowledge, there is no report describing the role of Rad in bone fracture healing and nonunion development.

As discussed here, Rad is highly expressed in the skeletal muscle of patients with type II diabetes [10]. Interestingly, clinical studies have suggested that patients with diabetes mellitus are more likely to experience complications during fracture healing, including delayed union and nonunion [26], to the extent that fracture healing is estimated to take approximately twice as long in patients with diabetes than in those without [27]. Tyndall et al. previously suggested that fracture healing may be impeded in these patients, since they exhibit decreased PDGF expression, which in turn, inhibits cell proliferation [28]. Consistent with this hypothesis, high TNF-α production has been reported in fracture calluses in animals with diabetes, and as shown by Kayal et al., high TNF-α levels both promote chondrocyte apoptosis and inhibit fracture healing [29]. As mentioned previously herein, TNF-α and PDGF have been reported to be involved in the regulation of *Rad* gene expression [11]. In the present study, *TNF-α* gene expression was significantly higher in the nonunions than in the standard healing fractures and the pattern of gene expression closely resembled that of *Rad*. In contrast, *PDGF* gene expression was significantly lower in the nonunions as overserved in the diabetic condition. These results suggested that the regulation of *Rad* gene expression levels in nonunions is associated with TNF-α but not with PDGF. Chronic inflammation, which involves potent pro-inflammatory cytokines such as TNF-α, is well known as a factor that can lead to the development of nonunion [30]. High concentrations of TNF-α have been reported to inhibit osteoblast differentiation [31], and one of the mechanisms is thought to be the downregulation of preosteoblast epidermal growth factor-like repeat protein by meprin, A5 protein, and receptor protein-tyrosine phosphatase l domain (POEM) [32]. We speculate that there is another unknown pathway by which TNF-α inhibits fracture healing via Rad upregulation. High levels of Rad are often observed in diabetic skeletal muscle; thus, elucidating this detailed mechanism may explain reasons for delayed fracture healing in patients with diabetes; however, more research is required to confirm the presence of this relationship.

The present study also investigated Rad and Rem1 protein expression (immunolocalization) in standard healing fractures and nonunions. Interestingly, while *Rad* and *Rem1* gene expression differed significantly in the standard healing fractures and nonunions, their protein expression patterns were very similar. For example, on post-fracture days 14 and 21, Rad and Rem1 in the standard healing fractures were detected at robust levels in fibroblast-like spindle cells, proliferating chondrocytes, and osteoblasts lining the trabecular bone, but only at very low levels in hypertrophic chondrocytes. Given that the differentiation of proliferating cells to hypertrophic chondrocytes is a vitally important process for endochondral ossification, this result suggests that the observed decrease in Rad expression may be associated with the progression of normal fracture healing process. This hypothesis was supported both by the real-time PCR results. As another possible explanation, because less hypertrophic chondrocytes, which seem to lack the expression of Rad, are present in the standard fracture than in the nonunion, the gene expression of Rad in the standard fracture might be lower than that in the nonunion.

Thus, results of the present study reveal that *Rad* expression patterns in standard healing fractures and nonunions are different from those of *Rem1*, despite their similar patterns of protein localization. This suggests that although both Rad and Rem1 are expressed in the fracture callus and fibrous tissues of standard healing fractures and nonunions respectively, their differential gene expression patterns may be reflective of their divergent roles in, and/or impacts on, nonunion development.

We have still some issues to be addressed. As one of the future directions, experiments investigating the effect of inhibiting PDGF and TNF-α using a Rad-overexpressing model should be considered. This might help to figure out how Rad affects nonunion development. Further, it is necessary to carry out investigations using a Rad-knock out model to determine how suppression of Rad influences the fracture healing process and nonunion development. As further in vitro experiment, studying the influences of Rad on MSC, osteoblasts, and chondrocytes would be helpful to understand the role of Rad in nonunion development. Furthermore, it would be necessary to conduct similar studies using female rats to determine whether Rad overexpression in nonunions occurs in only male rats. All of these are subjects of our future study. Rad is one of the genes or proteins that have not been fully explored and thus continuous research is necessary.

## Conclusion

This is the first study to investigate Rad and Rem1 gene and protein expression in both standard healing fractures and nonunions. The presented results suggest that Rad may be involved in nonunion development, and thus, may be a promising therapeutic target for nonunion treatment.

## Data Availability

The datasets used and/or analyzed during the current study are available from the corresponding author on reasonable request.

## Abbreviations

POEM: Preosteoblast epidermal growth factor-like repeat protein with meprin, A5 protein, and receptor protein-tyrosine phosphatase l domain
cDNA: complementary deoxyribonucleic acid
GAPDH: Glyceraldehyde-3-phosphate dehydrogenase
GTP: Guanosine triphosphate
K-wire: Kirschner wire
mRNA: messenger ribonucleic acid
PCR: Polymerase chain reaction
PDGF: Platelet-derived growth factor
Rad: Ras associated with diabetes
Rem1: Rad and gem related GTP binding protein 1
RNA: Ribonucleic acid
TNF-α: Tumor necrosis factor-α
